# Medical characteristics of the oldest old: retrospective chart review of patients aged 85+ in an academic primary care centre

**DOI:** 10.1186/1756-0500-7-340

**Published:** 2014-06-05

**Authors:** Christopher S Tsoi, Justin Y Chow, Kenny S Choi, Hiu-Wah Li, Jason X Nie, C Shawn Tracy, Li Wang, Ross EG Upshur

**Affiliations:** 1 Bridgepoint Active Healthcare, Toronto M4M 2B5, ON, Canada; 2 Cancer Care Ontario, Toronto M5G 2 L3, ON, Canada; 3 Faculty of Medicine, University of Toronto, Toronto M5S 1A8, ON, Canada; 4 Department of Family Medicine, Mount Sinai Hospital, Toronto M5T 3 L9, ON, Canada; 5 Department of Family and Community Medicine, Sunnybrook Health Sciences Centre, Toronto M4N 3 M5, ON, Canada; 6 Mount Sinai Hospital Sherman Health and Wellness Centre, 9600 Bathurst Street, Suite 300, Vaughan L6A3Z8, ON, Canada

**Keywords:** Primary care, Geriatrics, Multimorbidity

## Abstract

**Background:**

The population aged 85 + − the “oldest old” – is now the fastest growing age segment in Canada. Although existing research demonstrates high health services utilization and medication burden in this population, little clinically derived evidence is available to guide care. This is a descriptive study in a primary care context seeking to describe the most common health conditions and medications used in the “oldest old”.

**Methods:**

We conducted a retrospective chart review of all family practice patients aged 85+ (N = 564; 209 males, 355 females) at Sunnybrook Health Sciences Centre in Toronto, Canada. Electronic medical records were reviewed for all current chronic conditions and medication prescriptions, and then stratified by sex and age subgroup (85–89, 90–94, 95+) for descriptive analysis.

**Results:**

On average, patients experienced 6.4 concurrent chronic conditions and took 6.8 medications. Most conditions were related to cardiovascular (79%) and bone health (65%). Hypertension (65%) was the most common condition. Bone-related conditions (e.g. osteoarthritis, osteoporosis) and hypothyroidism predominantly affected women, while coronary artery disease and type 2 diabetes were more prevalent in men. The top two prescribed medications were atorvastatin (33%) and aspirin 81 mg (33%). Males were more likely to be prescribed lipid-lowering medications, while females were more likely to receive osteoporosis therapy. Patients received less lipid-lowering therapy with increasing age.

**Conclusions:**

Multimorbidity and polypharmacy are highly prevalent in patients in the 85+ age group. The most common clinical conditions are related to cardiovascular and bone health, and the most commonly prescribed medications are directed towards risk factors for these illnesses. In the absence of data to guide clinical decision-making, this study provides a first look at the common health concerns and medication profiles in this population and reveals trends that give rise to reflections on how clinical care for these patients can be improved.

## Background

The Canadian population is rapidly aging, with patients aged 85 + − the “oldest old” – now representing one of the fastest growing age segments in the country [1]. Compared to their 65-to-74-year-old counterparts, these patients have more chronic health conditions [2], use more health services [3] and have seen a dramatic rise in medication prescriptions [4]. Increased consumption of health services and polypharmacy are trends reflected in the concept of “multimorbidity”, which despite conflicting definitions is unequivocally more prevalent in patients aged 85+ than in any other age segment [5-7].

Much of the health care burden for this patient group falls on primary care providers [8]. As these providers seek to provide patient-centered and evidence-based care for this population, a major challenge lies in the lack of data describing even basic clinical characteristics of these patients as a group. Therefore, the primary objective of the present study is to determine the most prevalent health conditions and prescription medications among patients aged 85+ in an academic family practice setting. We will also describe the clinical complexity of these patients by using the “complexity score” [9] – a clinical tool to measure the extent of multimorbidity in the elderly. Our data reveals trends which give rise to reflections on how clinical care of the oldest old patients can be improved.

## Methods

### Ethics statement

Ethics approval was obtained prior to this study from the Sunnybrook Health Sciences Centre Research Ethics Office. This office specifically waived the requirement for written consent by the patients whose charts were reviewed.

### Study design

We conducted a retrospective chart review for all patients aged 85+ in the Family Practice Unit (FPU) at Sunnybrook Health Sciences Centre in Toronto, Canada.

### Setting and study population

The Sunnybrook FPU is an academic primary care practice fully-affiliated with the University of Toronto. The clinic has 13 staff family physicians that belong to an FPU team which also comprises medical trainees, nurses, social workers, dietitians and diabetes educators. The FPU provides care to approximately 8,500 patients, with a focus on older patients with complex chronic disease. All patients who were 85 years of age or older as of January 1, 2011 and had complete medical records (current chronic health conditions and prescribed medications) were included in this study (N = 564; 209 males and 355 females).

### Data collection

The electronic medical record of each eligible patient was reviewed and data was extracted from their Cumulative Patient Profile (CPP). The CPP is a section included in all Canadian electronic medical record systems [10] which allows family physicians to upkeep a patient’s information in a compact fashion, including his or her current chronic conditions and medications. However, the information is not standardized. We used an automated script to abstract each patient’s current chronic conditions and medication lists (CT) and manually reviewed the data for consistency and duplications (KC). Past medical history that was chronic in nature (e.g. hypertension, osteoarthritis) was counted as a current chronic condition.

### Analysis

Patients were grouped by sex and by 5-year age intervals (i.e. 85–89, 90–94, 95+) as determined by their age on January 1, 2011. Descriptive statistics for chronic illnesses and medications were calculated by sex and age group. For each patient, the “complexity score” was calculated by summing the number of long-term medications and the number of current chronic conditions. For example, a patient with five chronic conditions and three medications would be assigned a “complexity score” of eight. The “complexity score” is a measure that has been shown to have strong positive association with hospitalizations, emergency room visits, and family physician visits [9]. Patients were categorized by their complexity score into sextiles (i.e. 0–5, 6–10, 11–15, 16–20, 21–25, >25), and the distribution was studied against age and sex.

## Results

Table 1 shows the demographics of the study population by age group and sex. Overall, there were more females (63%) than males (37%). This difference in the distribution of sex widened with increasing age. The total number of patients in this study comprised 6.4% of the 8767 total patients in the family practice.

**Table 1:** Demographic profile of family practice patients aged 85+ at Sunnybrook Health Sciences Centre

	**85-89 yrs**	**90-94 yrs**	**95+ yrs**	**Total**
**Sex**				
Female	219 (61)	113 (65)	23 (72)	355 (63)
Male	140 (39)	60 (35)	9 (28)	209 (37)
Total	359 (100)	173 (100)	32 (100)	564 (100)

Values are numbers (percentages) of patients.

Table 2 presents data on the 10 most common medical conditions in our study sample overall and by sex. Overall, patients in our study sample experienced 6.4 chronic medical conditions (data not shown). Four of the 10 most common diseases were cardiovascular risk factors (hypertension, dyslipidemia, atrial fibrillation, type 2 diabetes) with hypertension the most common, prevalent in approximately 65% of the study population. Both males and females shared these 4 conditions in their top 10 list, along with non-cardiovascular conditions such as osteoarthritis and hearing loss. In our sample, osteoporosis, hypothyroidism, and gastro-esophageal reflux disease (GERD) were more common in females. Among males, coronary artery disease (16.3%) was more prevalent.

**Table 2:** Top 10 most common chronic conditions in patients aged 85+ by sex

	**Males**		**Females**		**Overall**	
**Rank**	**Disease**	**%**	**Disease**	**%**	**Disease**	**%**
1	Hypertension	61.7	Hypertension	66.8	Hypertension	64.9
2	Benign prostate hypertrophy	28.7	Osteoporosis	49.6	Osteoarthritis	37.1
3	Dyslipidemia	28.2	Osteoarthritis	42.5	Osteoporosis	35.8
4	Osteoarthritis	27.8	Dyslipidemia	23.4	Dyslipidemia	25.2
5	Atrial fibrillation	27.3	Hypothyroidism	21.7	Atrial fibrillation	22.7
6	Type 2 diabetes	22.0	Atrial fibrillation	20.0	Hypothyroidism	17.7
7	Chronic renal failure	18.2	Osteopenia	17.5	Type 2 diabetes	17.2
8	Hearing loss	16.8	Type 2 diabetes	14.4	Hearing loss	15.1
9	Coronary artery disease	16.3	GERD	14.4	Osteopenia	13.5
10	Glaucoma	14.4	Hearing loss	14.1	Glaucoma	13.3

Table 3 presents the top 10 most common diseases by age group. Across age groups it is apparent that the prevalence of hypertension, osteoarthritis, and osteoporosis increased with age. Conversely, dyslipidemia, diabetes, and coronary artery disease decreased in prevalence with advancing age and were not among the 10 most common conditions in the highest age bracket.

**Table 3:** Top 10 most common chronic conditions by age group

	**85-89 yrs**		**90-94 yrs**		**95+ yrs**	
**Rank**	**Disease**	**%**	**Disease**	**%**	**Disease**	**%**
1	Hypertension	63.2	Hypertension	67.1	Hypertension	71.9
2	Osteoarthritis	35.4	Osteoarthritis	38.7	Osteoporosis	65.6
3	Osteoporosis	32.0	Osteoporosis	38.2	Osteoarthritis	46.9
4	Dyslipidemia	27.6	Hypothyroidism	24.3	Atrial fibrillation	31.3
5	Atrial fibrillation	23.1	Dyslipidemia	22.5	GERD	25.0
6	Type 2 diabetes	18.9	Atrial fibrillation	20.2	Dementia (all types)	21.9
7	Hypothyroidism	15.0	Hearing loss	19.1	Hearing loss	21.9
8	Osteopenia	13.7	Glaucoma	16.2	Hiatus hernia	18.8
9	Degenerative disc disease	13.4	Type 2 diabetes	15.6	Depression	15.6
10	Coronary artery disease	12.5	Osteopenia	13.9	Anemia	12.5

Tables 4 and 5 present, respectively, the top 10 most common prescription medications and medication classes for each gender. Patients in our study were taking an average of 6.8 medications (data not shown). The range was 0 to 18 and the interquartile range was 5 to 9. Only 3 of the top 10 medication classes overall were meant for symptom relief, and these have been bolded in Table 5. The remaining 7 classes were prescribed for risk factor modification, including 4 for cardiovascular disease alone. Overall, 88.7% of patients were taking some type of cardiovascular medication. 207 out of the 337 patients (61.4%) who had been prescribed either aspirin, a statin, or both medications did not have a diagnosis of either coronary artery disease or Type 2 diabetes charted in their CPP (data not shown). There were no medications for glycemic control in the top 10 list for any gender or age group.

**Table 4:** Top 10 most common prescription medications in patients aged 85+ by sex

	**Males**		**Females**		**Overall**	
**Rank**	**Medication**	**%**	**Medication**	**%**	**Medication**	**%**
1	Atorvastatin	40.7	ASA (mini)	31.8	Atorvastatin	32.8
2	ASA (mini)	34.0	Atorvastatin	28.2	ASA (mini)	32.6
3	Ramipril	34.0	Hydrochlorothiazide	28.2	Hydrochlorothiazide	28.2
4	Hydrochlorothiazide	28.2	Levothyroxine	26.8	Ramipril	25.7
5	Metoprolol	26.3	Vitamin D	26.2	Amlodipine	23.8
6	Warfarin	24.9	Amlodipine	25.4	Vitamin D	23.4
7	Docusate	23.4	Alendronate	23.7	Levothyroxine	23.2
8	Amlodipine	21.1	Ramipril	20.9	Warfarin	20.7
9	Nitroglycerin spray	19.6	Acetaminophen	20.9	Docusate	20.6
10	Vitamin D	18.7	Calcium	20.9	Metoprolol	19.0

**Table 5:** Top 10 most common prescription medication classes in patients aged 85+ by sex

	**Males**		**Females**		**Overall**	
**Rank**	**Medication class**	**%**	**Medication class**	**%**	**Medication class**	**%**
1	Statins	59.8	**Analgesics, non-opioid**	**50.4**	**Analgesics, non-opioid**	**50.2**
2	**Analgesics, non-opioid**	**49.8**	Bisphosphonates	47.3	Statins	47.9
3	Beta-blockers	42.6	Statins	40.8	Beta-blockers	36.7
4	Ace-inhibitors	42.1	Calcium (alone or in combination with Vitamin D)	36.6	Bisphosphonates	34.4
5	Anti-thrombotic agents	35.9	CCBs	35.5	CCBs	34.0
6	**PPIs**	**35.4**	Beta-blockers	33.2	**PPIs**	**33.3**
7	**BPH drugs**	**35.4**	Thiazide diuretics (alone or in combination with other drugs)	32.7	Ace-inhibitors	33.3
8	CCBs	31.6	**PPIs**	**32.1**	Thiazide diuretics (alone or in combination with other drugs)	31.4
9	**Drugs for constipation**	**30.6**	**Drugs for constipation**	**30.4**	**Drugs for constipation**	**30.5**
10	Thiazide diuretics (alone or in combination with other drugs)	29.2	Ace-inhibitors	28.2	Calcium (alone or in combination with Vitamin D)	30.3

The bolded medications are for symptom relief. PPIs, proton pump inhibitors; BPH drugs, benign prostatic hypertrophy drugs; CCBs, calcium channel blockers.

Tables 6 and 7 present, respectively, the top 10 most common prescription medications and medication classes by age group. Across age groups, we found that the rate of statin use declined with advancing age and the use of acetaminophen rose. There was a decline in the number of different medications for cardiovascular risk factors (particularly hypertension) with increasing age.

**Table 6:** Top 10 most common prescription medications by age group

	**85-89 yrs**		**90-94 yrs**		**95+ yrs**	
**Rank**	**Medication**	**%**	**Medication**	**%**	**Medication**	**%**
1	Atorvastatin	37.3	Amlodipine	31.2	Amlodipine	31.3
2	ASA (mini)	34.5	Hydrochlorothiazide	30.1	Acetaminophen	31.3
3	Hydrochlorothiazide	28.4	ASA (mini)	29.5	ASA (mini)	28.1
4	Ramipril	26.5	Levothyroxine	29.5	Docusate	28.1
5	Vitamin D	25.4	Ramipril	27.2	Atorvastatin	25.0
6	Warfarin	22.3	Atorvastatin	24.9	Vitamin D	25.0
7	Levothyroxine	20.9	Docusate	24.3	Warfarin	25.0
8	Amlodipine	19.5	Vitamin D	19.1	Metoprolol	25.0
9	Metoprolol	19.2	Alendronate	17.9	Alendronate	21.9
10	Calcium	18.9	Acetaminophen	17.3	Calcium	21.9

**Table 7:** Top 10 most common prescription medication classes by age group

	**85-89 yrs**		**90-94 yrs**		**95+ yrs**	
**Rank**	**Medication class**	**%**	**Medication class**	**%**	**Medication class**	**%**
1	Statins	54.3	Analgesics, non-opioid	46.2	Analgesics, non-opioid	53.1
2	Analgesics, non-opioid	51.8	CCBs	43.4	Beta-blockers	40.6
3	Beta-blockers	37.3	Drugs for constipation	38.7	Bisphosphonates	40.6
4	Ace-inhibitors	34.5	Bisphosphonates	38.7	Drugs for constipation	37.5
5	PPIs	34.0	Statins	37.6	CCBs	37.5
6	Thiazide diuretics (alone or in combination with other drugs)	32.6	Beta-blockers	34.7	Calcium (alone or in combination with Vitamin D)	34.4
7	Bisphosphonates	31.8	Ace-inhibitors	34.7	Statins	31.3
8	Anti-thrombotic Agents	31.5	PPIs	32.9	Analgesics, opioids	31.3
9	Calcium (alone or in combination with Vitamin D)	31.2	Thiazide diuretics (alone or in combination with other drugs)	31.2	PPIs	28.1
10	CCBs	29.2	Thyroid therapy (hormone replacement or antithyroid)	30.1	Angiotensin II-antagonists, plain	28.1

The complexity scores followed a mildly skewed right normal distribution across age and sex categories. The mean and median complexity scores in our sample were 13.3 and 12.5, respectively. The range was 2 to 29. We did not observe any significant differences between categories. Approximately one-third of our sample (35%; n = 198) had a complexity score between 11–15.

## Discussion

This large study of community-dwelling seniors over the age of 85 demonstrates that this population is afflicted by a large number of common chronic diseases and takes a large number of medications. Hypertension, osteoarthritis, dyslipidemia, atrial fibrillation, type 2 diabetes and hearing loss are highly prevalent in both sexes. Other conditions show variance between genders, suggesting that patterns noted in younger age groups such as females with hypothyroidism [11] and males with coronary artery disease [12] persist into older age.

The study of disease prevalence between age brackets in our data set also reveals insights that are relevant to the clinical care of younger seniors. Specifically, there seems to be a survivor effect for conditions such as dyslipidemia, diabetes, and coronary artery disease in that the patients who survived the longest were the least likely to be afflicted by these conditions. Such information may be valuable in conversations with patients about clinical decisions such as the continuance of cancer screening into age ranges that are not addressed in clinical practice guidelines, as the usefulness of such preventive health manoeuvres is affected by the patient’s expected remaining lifespan. These types of conversations are becoming increasingly common in Canada with the rapidly growing senior cohort and the establishment of cancer screening programmes.

Many of the medications prescribed to patients in this study were for risk modification. Of the top 10 most frequently prescribed medication classes overall, only 3 (non-opioid analgesics, proton pump inhibitors, and drugs for constipation) were aimed at symptom relief. A similar pattern has been documented at the population level [4].

The extent of this disproportion is made more conspicuous by the fact that in an era where scientific evidence is so often cited to justify medical practice, there is a total lack of evidence in this age group to support the efficacy of the risk-modifying medications that are being prescribed with such frequency.

Furthermore, well-intentioned efforts to extend the length of life through risk modification may ironically result in worsening the quality of life in complex elderly patients. Other authors [13] have written about how patients can become confused and their daily schedules overtaken by complicated medication regimens resulting from the implementation of multiple clinical practice guidelines which may even contradict each other. Quality of life also suffers when patients experience medication adverse effects, which are more likely in the setting of polypharmacy [14].

There are some encouraging trends in our data which illustrate practical responses to the concerns outlined above. Firstly, while there are no risk-modifying medications shown to have benefit in this old population, there are some which may cause harm. These should be used with great caution. For example, diabetes medications can cause hypoglycemic episodes in the elderly [15] and our data indicate that the physicians whose patients we studied did not prescribe these medications frequently.

Secondly, while there is no evidence directly in the oldest old for risk-modifying medications, there are trends in the data from younger age groups which can be used to inform thoughtful prescribing decisions for their older counterparts. For example, it has been observed that the time to benefit [16] for many of these medications is often measured in years, and thus it is rational to use them less often as patients age and have fewer expected years of life remaining. In our data set, this principle is reflected in the decreasing use of statin medications with increasing age and the infrequent use of anti-hyperglycemics.

There was no difference in complexity scores between age or sex categories, but the overall median score of 12.5 indicates a high level of complexity in this patient population. This translates into a more than 2-fold potential increase in hospitalization and emergency department admissions compared to patients with a complexity score between 0–5 [9]. Although the mean complexity score in this patient sample was high, the distribution of scores was wide (ranging from 2 to 29). This reflects the heterogeneous nature of even the oldest old population and serves as a useful reminder for the clinician that they must not be viewed through a “one size fits all” lens. The most complex patients take a large number of medications and are at high risk for drug interactions, and thus it is even more difficult to justify further complicating their regimen with additional unproven risk-modifying medications as discussed above. Conversely, there are patients over 85 years old who have low clinical complexity and excellent quality of life who wish to take these medications even while recognizing the lack of data derived from subjects their age, because there is a chance that they will benefit and live longer as a result. Such patient preferences should be respected. In fact, recent guidelines [17] have made explicit this very idea of a patient-centred approach to risk modification that accounts for a patient's clinical status.

The major strength of this study is the large number of patients 85 years of age and older who were included.

This study is subject to certain limitations. The sample of patients came from a single primary care unit in an academic health centre in Toronto, located in an educated and affluent neighborhood. Each of these traits may have an influence on the ability to generalize our results to other seniors over the age of 85. For example, the high rate of osteoporosis may be due to the fact that in an academic environment, great emphasis was placed upon adhering to screening guidelines. It has been shown elsewhere that poor socioeconomic status (SES) places one at higher risk for coronary artery disease [18], and thus the relatively low rate of CAD in our study may be partially explained by the clinic’s location in a high SES neighborhood.

The study was also limited because its data was extracted exclusively from the CPP. Each patient's physician is responsible for the upkeep of this portion of the patient chart, and therefore variation in their charting habits could have influenced our results. For example, the disparity between the prevalence of osteoporosis in females and prescriptions of calcium and Vitamin D may be due to the fact that clinicians recommended that their patients buy these supplements over-the-counter, and did not record this in their medication list. It is also likely that we missed chronic conditions which were not entered in the CPP despite being documented in the body of patients’ charts.

## Conclusions and recommendations

As the aging Canadian population expands and their burden of health care falls largely on the shoulders of family physicians, it is becoming ever more important to understand the health characteristics and medication profiles of the oldest old. Our study demonstrates that 6.4% of the patients in a family practice are above 85 years old, and that the majority of documented chronic conditions in this population are related to both cardiovascular and bone health. The data also highlights the significant number of medications used on a daily basis by older adults. This information provides the basis for reflection on several issues relevant to the clinical care of the oldest old such as the necessity of risk-modifying medications, the prioritization of quality versus quantity of life, and the importance of recognizing heterogeneity in this population.

## Competing interests

The authors declare that they have no competing interests.

## Authors’ contributions

CT performed the data extraction from patient charts, wrote the first draft of the manuscript, and consolidated the valuable contributions of other authors. KC reviewed the extracted data and standardized it. JC and HL analyzed the data and contributed to the manuscript. JN, ST, and LW contributed to the manuscript. RU conceived the idea for the study and contributed to the manuscript. All authors read and approved the final manuscript.
